# Social Determinants of Health in Patients Undergoing Osteocutaneous Free Flap Reconstruction for Malignant Disease of the Mandible

**DOI:** 10.1002/hed.28218

**Published:** 2025-06-17

**Authors:** Rohith M. Bhethanabotla, Nina Patel, Reta Behnam, W. John Boscardin, Mary J. Xu, Chase Heaton, Katherine C. Wai, Andrea M. Park, P. Daniel Knott

**Affiliations:** ^1^ Department of Otolaryngology—Head and Neck Surgery University of California—San Francisco San Francisco California USA; ^2^ University of California—San Francisco School of Medicine San Francisco California USA; ^3^ Department of Medicine and Epidemiology and Biostatistics University of California—San Francisco San Francisco California USA

**Keywords:** health equity, mandibular reconstruction, osteocutaneous free flap, social determinants of health, surgical outcomes

## Abstract

**Background:**

Social determinants of health (SDH), such as race, language, and insurance status, may impact access to surgical care and postoperative outcomes. This study investigates the role of SDH in patients undergoing osteocutaneous free flap reconstruction for mandibular defects due to oral cavity malignancy.

**Methods:**

A retrospective cohort study of 131 patients at a single tertiary academic center was conducted. Demographic variables and clinical outcomes were extracted from records. The CDC Social Vulnerability Index (SVI) assessed county‐level SDH. Outcomes included readmission, reoperation, flap failure, tracheostomy outcomes, and complications.

**Results:**

No significant differences were observed in clinical outcomes based on race, language, or insurance status. The only significant difference was travel distance, with non‐minoritized patients traveling farther (84.7 vs. 44.2 miles, *p* = 0.013). SDH metrics did not influence surgical timing or complications.

**Conclusions:**

In a well‐supported care setting, equitable access to complex reconstructive surgery may reduce disparities associated with SDH.

## 1 Introduction

Osseous involvement of the mandible among patients with oral cavity malignancy ranges between 12% and 56% [1, 2, 3, 4, 5]. Osteocutaneous free flap reconstruction is almost exclusively employed to restore both functionality and aesthetics in these patients. However, resection with osseous microvascular reconstruction carries significant perioperative risks, necessitates the collaboration of multiple care teams, and requires extensive postoperative follow‐up, rehabilitation, and support services [3, 6, 7, 8, 9, 10, 11, 12]. Therefore, patient recovery is heavily dependent on timely access to high‐quality care, which can be profoundly influenced by a continuum of social and environmental conditions—factors that often manifest beyond the clinic walls.

Social determinants of health (SDH) refer to the vast array of economic, legal, political, social, and environmental conditions that shape health outcomes, aside from biological bases of disease [13]. These factors encompass race, ethnicity, gender, sexual orientation, socioeconomic status, neighborhood safety, access to education, and housing stability, among others that can collectively shape an individual's health risks, opportunities, and outcomes [14, 15]. SDH are critical considerations for healthcare providers and health systems in the pursuit of high‐quality healthcare delivery as they reflect the structural barriers and systemic injustices faced by marginalized communities [13, 14, 15]. While SDH have been well documented in the context of chronic diseases such as diabetes and cardiovascular disease—where disadvantaged populations experience higher rates of morbidity and delays in care—there remains a paucity of research related to how SDH may affect outcomes within surgical subspecialties [16, 17]. This gap in the existing literature underscores the need to better understand whether patients with higher vulnerability profiles experience higher complication rates, prolonged hospital stays, and worse functional outcomes following reconstructive surgery for oral cavity cancers.

At present, studies have investigated how head and neck cancers (HNCs) themselves are intimately tied to SDH, with vulnerable populations more likely to be younger, not receive human papillomavirus (HPV) vaccination, misuse tobacco and alcohol, live in poverty, and possess lower health literacy [18]. The culmination of these factors contributes to higher morbidity and mortality rates, while also presenting challenges to surgery [13, 19]. They are associated with delayed diagnoses, longer operating times, increased complications, and lower adherence to post‐treatment surveillance [13, 18, 20]. Building on this foundational understanding, our study explores how SDH influences outcomes for patients undergoing free flap reconstruction for mandibular defects at our tertiary care center. We selected this population because patients requiring osseous free flap reconstruction for oral cavity malignancy represent a highly complex and vulnerable surgical cohort. These cases demand multidisciplinary coordination, complex morbidities, prolonged hospitalization, and extensive follow‐up. As such, this population serves as an effective lens and ideal “stress test” to evaluate whether institutional infrastructure and coordinated care pathways can buffer against disparities related to SDH. Utilizing an existing institutional free flap database, we aim to provide detailed insights into patient‐ and surgery‐specific outcomes which are often unavailable in larger national datasets.

## 2 Methods

This study was approved by the Institutional Review Board at the University of California, San Francisco.

### 2.1 Data Collection

A retrospective chart review of electronic medical records was conducted for all patients diagnosed with Stage IV oral cavity malignancy involving the mandible, requiring mandibulectomy and osteocutaneous free flap reconstruction between January 2011 and December 2023. Exclusion criteria included patients with benign mandibular tumors, nonneoplastic causes for mandible reconstruction (e.g., osteoradionecrosis), prior free flap reconstruction or radiation therapy in the head and neck region, out‐of‐state residency, age under 18, pregnancy, or incarceration at the time of treatment to ensure a homogenous cohort without possible confounding variables.

Extracted data included demographics (e.g., age, gender, race, and zip code) as well as medical and social history (e.g., insurance status, tobacco use). Driving distances were calculated based on patients' residential and surgical center addresses. Preoperative comorbidity status was assessed using the Charlson Comorbidity Index (CCI) and categorized as mild, moderate, or severe [21]. Zip code variables (e.g., median income, unemployment rate, poverty rate, percentile of individuals with English as primary language, and percentage of minority individuals) were derived from the US Census Bureau database [22]. Overall social vulnerability was assessed using the CDC's 2022 nationwide Social Vulnerability Index (SVI) by County [23].

Surgical data were extracted from an existing institutional free‐flap database. Variables included dates of initial consultations with ablative and reconstructive surgeons, surgery date, 30‐day hospital readmission rates, reoperation rates, incidence of partial or total flap failure, time to tracheostomy decannulation, requirement for tracheostomy at discharge, and 30‐day postoperative complications. Medical complications were defined as pneumonia, myocardial infarction, non‐MI cardiac events (e.g., heart failure, arrhythmias, nonspecific chest pain), pulmonary embolism, deep venous thrombosis, and sepsis. Thirty‐day postoperative complications included infections at donor or recipient sites, wound dehiscence, fistula formation, flap loss, and hematoma.

### 2.2 Statistical Analysis

Statistical analysis was performed using Stata (version 17.1, StataCorp LLC, College Station, TX). Descriptive statistics characterized patient demographics, comorbidities, and perioperative findings. Numerical variables are reported as means with standard deviations (SDs), and categorical variables as percentages. Univariate analysis compared data from two groups: patients from non‐minoritized groups who self‐identify as non‐Hispanic White versus patients from minoritized groups represented from Hispanic White, non‐Hispanic Black or African American, Asian, Native Hawaiian or Pacific Islander, and American Indian or Alaska Native groups. Patient race and ethnicity data was inputted into respective electronic medical records from self‐reported intake forms prior to data extraction for this study. Groups were dichotomized due to close to equal sample sizes. Spearman's correlation analysis examined relationships between time from reconstructive consultation to surgery, hospital length of stay, and total complications with SDH metrics, including age, income, driving distance, and county‐level indicators. Multivariable regression models were constructed with dichotomized SDH variables to facilitate interpretation. Statistical significance was set at a two‐tailed *p* value of < 0.05.

## 3 Results

### 3.1 Entire Patient Cohort

One hundred thirty‐one patients were included in the analysis, summarized in Table 1. The cohort predominantly comprised non‐Hispanic men (55.0%) identifying as White (53.4%), with a median age of 64 years (IQR: 56–73). Most were English‐speaking (82.4%) and insured (97.7%). Preoperative comorbidities were classified as severe (40.8%) or moderate (47.7%), and smoking history included never (46.2%) or former smokers (46.2%).

**TABLE 1:** Patient demographics.

Characteristics	Mean (SD) or number of patients (%)
Total patients	131
Race/ethnicity	
Hispanic	15 (11.5%)
Non‐Hispanic White	70 (53.4%)
Non‐Hispanic Black	9 (6.9%)
Non‐Hispanic Asian	34 (26.0%)
Native Hawaiian/Pacific Islander	2 (1.5%)
Other	1 (0.8%)
Gender	
Women	59 (45.0%)
Men	72 (55.0%)
Age (years)	63.8 (13.2)
Primary language	
English speaking	108 (82.4%)
Non‐English speaking	23 (17.6%)
Insurance	
Uninsured	3 (2.3%)
Private insurance	39 (29.7%)
Public insurance	89 (68.0%)
Household income by zip code	$105636.50 ($46651.80)
Distance to hospital (miles)	66.0 (71.4)
Comorbidities	
Preoperative Charlson Comorbidity Index	
Mild	15 (11.5%)
Moderate	53 (40.8%)
Severe	62 (47.7%)
Smoking status	
Current	10 (7.7%)
Former	60 (46.2%)
Never	60 (46.2%)
Free flap type	
Osteocutaneous radial forearm	6 (4.6%)
Fibula	125 (95.4%)

Zip code data showed an average household income of $105 636.50 with a SD of $46 651.80 and an average travel distance of 66.0 miles (SD: 71.4) from the hospital. All patients had advanced‐stage oral cavity cancer involving the mandible, and the most common flap type was fibula (95.4%). Patients originated from 27 counties, primarily in the San Francisco Bay Area, Northern California, and Central California. Based on the SVI‐CDC database, 53 patients (40.7%) resided in areas of low social vulnerability, 40 (30.7%) in low‐medium, 27 (20.8%) in medium‐high, and 10 (7.8%) in high social vulnerability areas.

Patients most often initially consulted with the ablative surgeon for oncologic treatment, followed by a consultation with the reconstructive surgeon. The time from ablative surgeon consultation to surgery was 35.9 days (34.2), and from reconstructive surgeon consultation to surgery was 14.8 days (16.7). Virtual surgical planning for patient‐specific mandibular guides was utilized in 36 patients (28.8%). A total of 61 complications were reported within 30 days—27 medical and 34 surgical (Table 2). Pneumonia (12 patients, 44.4%) was the most common medical complication, while donor/recipient site infections (9 patients, 26.5%) were the most common postoperative complications. Overall flap survival was 100%, with 4 cases of partial failure. The average length of stay was 10.8 days (SD: 6.2), including 3.2 days (SD: 2.8) in the ICU and 7.6 days (SD: 5.4) on the wards. Average time to tracheostomy decannulation was 8.1 days (SD: 3.7), and 8 patients (6.5%) required tracheostomy at discharge.

**TABLE 2:** Descriptive statistics of complications.

Complications	Number of complications	Percentage of complication type (medical or surgical, %)	Percentage of total complications (%)
*Medical complications*	27	100.0	44.3
Pneumonia	12	44.4	19.7
Non‐MI cardiac complications	11	40.7	18.0
Sepsis	2	7.4	3.3
Deep venous thrombosis	1	3.7	1.6
Pulmonary embolism	1	3.7	1.6
Myocardial infarction	0	0.0	0.0
*Postoperative complications*	34	100.0	55.7
Wound infection	9	26.5	14.8
Wound dehiscence	8	23.5	13.1
Hematoma	7	20.6	11.5
Fistula	4	11.7	6.6
Partial flap failure	3	8.8	4.9
Other	3	8.8	4.9
Total flap failure	0	0.0	0.0

### 3.2 Correlational and Multivariate Analysis

Spearman correlations analyzed associations between county‐level SVI‐CDC variables and outcomes, including time to surgery, length of stay, and complications (Table 3). No statistically significant correlations were found. Multivariate logistic models further assessed these relationships. Continuous variables were dichotomized for analysis (e.g., income > $100 000, age > 70, distance > 50 miles). Categorical variables (e.g., minoritized vs. non‐minoritized, men vs. women, English‐speaking vs. non‐English‐speaking) were already dichotomized. No statistically significant associations were identified.

**TABLE 3:** Comparison of minoritized versus non‐minoritized patients.

	Non‐minoritized		Minoritized		*p*
	*N*	%	*N*	%	
Number of patients	70	53.4	61	46.6	N/A
Gender					0.385
Women	29	41.4	30	49.2	
Men	41	58.6	31	50.8	
Age					0.410
< 70 years old	46	65.7	40	65.6	
≥ 70 years old	24	34.3	21	34.5	
Primary language					**< 0.001**
English‐speaking	70	100	38	62.3	
Non‐English speaking	0	0	23	37.7	
Insurance status					0.292
Uninsured	3	4.3	0	0.0	
State or private insurance	67	95.7	61	100.0	

	Mean	SD	Mean	SD	
Household income by zip code	$105 305.40	$48 880.20	$106 023.70	$44 294.60	0.651
Distance traveled	84.7	87.6	44.9	37.5	**0.013**
Time from ablative surgeon clinic to surgery	36.3	39.0	35.5	27.9	0.932
Time from reconstructive surgeon clinic to surgery	15.0	17.3	14.6	16.0	0.875
ICU length of stay	3.3	2.7	3.1	2.8	0.535
Ward length of stay	7.3	4.5	8.0	6.4	0.922
Time to PO intake	30.2	28.3	39.2	139.3	0.608
Time to decannulation	8.2	4.1	7.9	3.3	

	*N*	%	*N*	%	
Discharged with tracheostomy	6	9.2	2	3.4	0.279
Readmission within 30 days	7	10.1	7	11.9	0.783
Reoperation within 30 days	9	13.0	7	11.9	1.000

*Note:* Significant values are noted in bold text.

### 3.3 Comparison of Patients in Minoritized Versus Non‐Minoritized Groups

Additional univariate analysis assessed racial differences in access and outcomes (Table 4). Patients from minoritized groups (61, 46.6%) included 31 men (50.8%) and 30 women (49.2%). All non‐English speakers self‐identified as non‐White. The only significant difference was distance traveled; patients from non‐minoritized groups traveled farther (84.7 miles [SD: 87.6]) than patients from minoritized groups (44.9 miles [SD: 37.5]) (*p* = 0.013). Demographics, including gender, age, language, insurance, income, and outcomes such as surgery wait time, length of stay, oral intake initiation, decannulation timing, tracheostomy rates, readmission, reoperation, and complication rates, showed no significant differences between groups.

**TABLE 4:** Correlation analysis.

Predictor	Rho	*p*
*Correlating to time to surgery after reconstruction surgeon consultation*		
Age	0.041	0.648
Income by zip code	0.077	0.389
Distance traveled	−0.159	0.075
Percentile poverty by zip code	0.088	0.326
Percentile unemployment by zip code	0.065	0.464
Percentile uninsured by zip code	−0.041	0.649
Percentile non‐English primary language by zip code	−0.017	0.844
Percentile of minority residents by zip code	−0.017	0.847
*Correlating to total length of stay*		
Age	0.045	0.610
Income by zip code	−0.025	0.777
Distance traveled	−0.092	0.304
Percentile poverty by zip code	−0.063	0.478
Percentile unemployment by zip code	−0.017	0.849
Percentile uninsured by zip code	−0.049	0.585
Percentile non‐English primary language by zip code	−0.049	0.585
Percentile of minority residents by zip code	0.079	0.375
*Correlating to total complications per patient*		
Age	0.070	0.436
Income by zip code	0.131	0.143
Distance traveled	−0.148	0.097
Percentile poverty by zip code	−0.084	0.344
Percentile unemployment by zip code	−0.064	0.477
Percentile uninsured by zip code	−0.055	0.535
Percentile non‐English primary language by zip code	0.088	0.325
Percentile of minority residents by zip code	0.149	0.094

## 4 Discussion

This study is the first to describe associations of various indices of social vulnerability with variables that reflect access to and outcomes of complex HNC reconstructive surgery, particularly osteocutaneous free flaps of oral cavity cancer. Herein, we employed a comprehensive SDH index based on precise data from the US Census and we utilized our institution's dedicated free flap database, which allowed us to capture high‐resolution, patient‐specific, and flap‐specific outcomes that are not typically available in the existing national databases. We believe our research described can serve as a model for other institutions to investigate biases within their HNC treatment pathways and develop individualized, tailored programs that meet the needs of their unique communities.

Within this cohort, markers of social vulnerability, such as minority status, insurance status, primary language, and zip‐code–level socioeconomic indicators, were not significantly associated with differences in access to care or short‐term surgical outcomes. These findings reflect relative differences within a high‐acuity population, rather than a comparison between socially vulnerable and non‐vulnerable patients. The high overall flap survival rate, low complication rates, and comparable consultation‐to‐surgery times and hospital stays emphasize that sociodemographic disparities in clinical outcomes were largely mitigated within our tertiary academic care center. This suggests that robust institutional infrastructure and prioritization of late‐stage oncologic cases can play a critical role in ensuring equitable access to high‐quality surgical care.

Our cohort predominantly included patients with moderate‐to‐severe comorbidities based on CCI groupings, and more than half were current or former smokers—factors consistent with established risk profiles for HNC [24]. However, our cohort differed from national trends, with a nearly 1:1 male‐to‐female ratio, a lower representation of non‐Hispanic Black self‐identifying patients, and higher average household incomes compared to the United States at large [25, 26].

At present, HNC occurs more frequently in men than in women with an incidence ratio of 3:1 [27, 28, 29]. The higher proportion of women in our study, consequently, may have contributed to gender‐based differences in healthcare utilization; women are more likely to seek medical attention earlier, advocate for care and proactively schedule appointments, and adhere to treatment plans—factors that may ultimately improve outcomes [30]. Additionally, our cohort's disproportionately high representation of Asian identifying patients and lower representation of Black patients raises important questions about racial disparities. While Asian patients tend to have better survival rates for HNC, Black patients often present with more advanced disease and decreased survival compared to White patients with oral cavity cancers, despite comparable rates of smoking [31, 32, 33]. These cohort differences, which may reflect regional demographics unique to California, highlight the importance of future studies evaluating geographic and systemic influences on access to care and outcomes.

Our department's emphasis on holistic care through interprofessional collaboration likely contributed to equitable outcomes. As part of our workflow, patients are screened early for adverse SDH by both the ablative and reconstructive surgery teams. They are then referred based on a positive screen and triaged based on urgency to dedicated HNC social workers and patient navigation services, who possess extensive knowledge of local county resources, transportation assistance, and lodging options. Within our cohort, nearly 20% of patients spoke a primary language other than English. To address this, our scheduling services provide multilingual support, which likely improved communication, fostered trust, and enhanced patient engagement with the healthcare team. Finally, the integration of telehealth for postoperative follow‐ups allows patients traveling from distant areas to maintain regular access to medical care. Our results demonstrate that despite significant differences in travel distances between minoritized and non‐minoritized patients, there were no statistical differences in operative outcomes.

In our study, we performed an in‐depth analysis on the differences in care between patients in ethnic or racial minoritized groups versus patients from historically non‐minoritized backgrounds. Race plays a critical role as a variable in SDH due to its longstanding association with systemic disparities in healthcare. While race is a social construct, its effects on health outcomes often reflect the compounded influence of SDH, including barriers to care, unequal resource distribution, and historical practices such as segregation and redlining [34, 35, 36]. These structural factors continue to shape access to medical services, preventive care, and health literacy, perpetuating disparities along racial and socioeconomic lines. Despite the importance of accounting for race in similar health research, recent studies have revealed gaps in how these data are collected, with nearly half of HNC studies over the last decade failing to document study participants' race or ethnicity accurately [37]. This oversight may obscure meaningful patterns in race‐based disparities, limiting efforts to address inequities effectively. Given these concerns, we found it particularly important to incorporate race as a variable to perform further analysis on to better understand the intersection of socioeconomic and structural factors influencing health outcomes.

Despite its strengths, our study has several limitations. In performing an analysis based on racial and ethnic subgroups, our relatively small sample size limited the statistical power to detect smaller but potentially clinically meaningful differences within subgroups, requiring us to compare patients as minoritized versus non‐minoritized. It remains critical to analyze individual racial and ethnic subgroups due to the heterogeneity within these populations, as broad categories can mask significant variations in health outcomes, socioeconomic conditions, and healthcare access. The retrospective design inherently limits the ability to control for unmeasured confounders and biases. Additionally, the single institution focus, while beneficial for data consistency, may restrict generalizability to other healthcare systems with fewer resources or differing patient demographics. In this study, we excluded out‐of‐state patients to ensure uniformity in referral mechanisms, insurance structures, and follow‐up care protocols, as these individuals often access tertiary centers through non‐standard pathways and may receive portions of their postoperative care elsewhere. This exclusion allowed us to reduce confounding related to variability in care continuity and resource utilization. However, it may have also eliminated a potentially informative comparison group. Out‐of‐state patients are often more financially or logistically resourced, and their inclusion could have provided a contrast to better evaluate the impact of SDH. Finally, the use of zip‐code‐level data to approximate socioeconomic factors, rather than direct patient‐reported measures, may also introduce misclassification or ecological bias, as it may not accurately reflect individual patients' socioeconomic status or account for cost‐of‐living variability within zip codes across California.

Given the uniformly high‐acuity nature of our cohort, it is possible that patients shared overlapping vulnerabilities, limiting the contrast between social risk profiles. As such, our findings should be interpreted as reflecting relative outcome parity within a vulnerable population, rather than the elimination of disparities.

Future research should incorporate multi‐center studies to assess whether these findings are replicable in different practice settings with more heterogeneous populations, including rural and community hospitals with fewer resources. Larger sample size studies may allow for investigation of various racial and ethnic categories to better understand how SDH may differ across groups. Evaluating long‐term functional outcomes, quality of life, and patient‐reported barriers to care will further enhance our understanding of SDH impacts. Additionally, integrating prospective designs and qualitative assessments can help identify subtle, nuanced barriers that retrospective studies may overlook.

## 5 Conclusion

This study highlights that a multidisciplinary care environment in an academic tertiary center can mitigate disparities related to SDH in patients undergoing osteocutaneous free flap reconstruction for oral cavity malignancies. We found no significant differences in postoperative outcomes, complications, or access to care based on race, income, language, or insurance status. The only disparity observed was longer travel distances for non‐minoritized patients, which did not affect surgical outcomes. The findings described herein underscore the importance of coordinated workflows, early adverse SDH screening, and support services like patient navigation and multilingual resources in improving equitable care delivery. Incorporating technology like mobile health portals and telehealth follow‐ups may further optimize access and outcomes for distant patients. While our results are encouraging, limitations include the retrospective design, single‐institution focus, differences in cohort demographics due to region, and reliance on zip‐code‐level socioeconomic data that could introduce bias. Further research should validate these findings across diverse settings and assess longer‐term outcomes.

## Conflicts of Interest

The authors declare no conflicts of interest.

## Data Availability

The data that supports the findings of this study are available in Supporting Information of this article.
